# Long-term mortality and hospitalisation among Italian veterans deployed to the Balkans: a retrospective longitudinal study based on individual linkage with national health data

**DOI:** 10.3389/fpubh.2025.1605457

**Published:** 2025-09-19

**Authors:** Roberta De Angelis, Silvia Rossi, Marcella Bugani, Alberto Autore, Marco Lastilla, Raffaele Vento, Anna Rocchetti, Stefano Guzzinati, Massimo Vicentini, Valerio Manno, Lorenzo Spizzichino, Serena Battilomo, Simona Belmonte, Mauro Biffoni, Alfonso Piciocchi

**Affiliations:** ^1^Department of Oncology and Molecular Medicine, Istituto Superiore di Sanità, Rome, Italy; ^2^Defence General Staff, General Inspectorate of the Military Health Services, Rome, Italy; ^3^Veneto Tumour Registry, Epidemiological Department, Azienda Zero, Padova, Italy; ^4^Epidemiology Unit, AUSL-IRCCS di Reggio Emilia, Reggio Emilia, Italy; ^5^Service of Statistics, Istituto Superiore di Sanità, Rome, Italy; ^6^Directorate of Health Prevention, Ministry of Health, Rome, Italy; ^7^Directorate of Digitalisation, Health Information System and Statistics, Ministry of Health, Rome, Italy; ^8^GIMEMA Foundation, Rome, Italy

**Keywords:** occupational medicine, environmental health, hospitalisation, mortality, veterans, Balkans, neoplasms, depleted uranium

## Abstract

**Introduction:**

The potential health risks among veterans deployed to the Balkans have long been debated internationally. In Italy concern about cancer risk in veterans deployed to the Balkans in 1995–2004 (SEBAL cohort) originated from an initial study reporting an excess risk of Hodgkin’s lymphoma. Subsequent studies have not fully dispelled these concerns due to methodological shortcomings and the lack of a national cancer registry. Our aim is to investigate long-term mortality and hospitalisation risk in SEBAL veterans for cancer and other major pathologies in comparison to the general population and to non-deployed military peers.

**Methods:**

By linking with national archives of causes of death (1999–2018) and hospital discharges (2005–2018), long-term mortality (20 years) and hospitalisation (14 years) of the SEBAL cohort (71,144 soldiers) were evaluated, for the first time, for major disease groups and 27 cancer types. Standardized Mortality Ratios (SMR) and Standardized Hospitalisation Ratios (SHR) were used to compare health outcomes versus the Italian population (IP) and a military cohort never deployed abroad (114,260 Carabinieri).

**Results:**

The SEBAL cohort showed a lower risk of death and hospitalisation than both control populations for all natural causes (SMR = 0.40 and SHR = 0.80 vs. IP; SMR = 0.72 and SHR = 0.80 vs. Carabinieri), infectious, endocrine, respiratory, circulatory, digestive and genitourinary diseases, with the only exception of a significantly higher hospitalisation risk for musculoskeletal diseases vs. IP (SHR = 1.27). Reduced risks of hospitalisation and death were found for all cancers (SHR = 0.82 and SMR = 0.56 vs. IP; SHR = 0.91 and SMR = 0.78 vs. Carabinieri), head and neck, respiratory and digestive cancers. Marginally increased hospitalisation risks vs. IP, with no effect on mortality, were observed for thyroid cancer (+39%) and skin melanoma (+32%). For all other solid cancers, hospitalisation and death risks were lower or not significantly different. We found no increased risk for hematological neoplasms, including Hodgkin’s lymphoma.

**Conclusion:**

Overall, the results of this observational study of unprecedented follow-up and completeness do not support specific long-term risks of mortality or hospitalisation linked to peacekeeping missions to the Balkans.

## 1 Introduction

The potential health risks faced by military personnel deployed to conflict zones have long been a subject of concern and debate. Concern was primarily associated with the use of depleted uranium (DU) munitions on the battlefields, which has raised questions about potential exposure and subsequent long-term health effects. Inalation of impact aereosol and dust are recognized as a route of exposure for military personnel operating in areas contaminated by DU (1). This exposure is due to the chemical toxicity of DU and the internal exposure to alpha and beta radioactivity (2–4). Overexposure to DU has been associated to many putative risks for human health, such as kidney and bone diseases (5), leukemias/lymphomas, lung, bladder, and testicular cancers (6).

The debate surrounding this issue has led to specific studies aimed at assessing the excess risk of cancer in military personnel deployed to war theatres during the 1990s and 2000s. These studies initially focused on US and UK veterans of the Gulf War (7–15) and subsequently extended to include veterans from Sweden, Denmark, Canada, Italy, the Netherlands, Norway, and Finland who served in peacekeeping missions in the Balkans region (16–22).

The Italian Armed Forces have played a significant role in peacekeeping operations in Bosnia and Kosovo since 1995. A committee appointed by the Italian Ministry of Defense reported (23) an excess of Hodgkin’s lymphoma cases diagnosed between 1996 and 2001 but found no increased risk of other types of cancer among Italian veterans.

Subsequent epidemiological studies on cancer incidence in the Balkan veterans confirmed lower cancer risk compared to the Italian population and the significant excess of Hodgkin’s lymphoma, concentrated mainly in the year 2000 (21, 24, 25). However, these studies suffered from structural under-reporting for discharged personnel in the military health surveillance databases, which limited the exhaustive detection of cancer incidence (26, 27).

The risk of death among Balkan veterans was comprehensively assessed by cross-referencing national mortality data in the first SEBAL (Epidemiological Surveillance of the Balkan veterans) study (28). The study ascertained vital status during the period 1995–2008 and found no excess risk of death associated with Balkan deployment compared to the Italian population and to a military control population. Cancer death risk was also lower than expected in the Italian population. This study had some limitations, particularly a short follow-up (compared to the long latency implied in carcinogenesis processes) and the use of mortality outcome (not a sufficiently sensitive endpoint to study cancers with good prognosis).

Identifying post-deployment cancers among the veterans is a viable approach in countries with national cancer registries (17–20). In Italy this is not feasible, given the lack of a national registry and the uneven rollout of regional cancer registries both territorially and in terms of time.

To address the limitations of previous studies, the present study extends the follow-up period of the first SEBAL study by 10 years and incorporates a morbidity study based on hospital discharge records, which are available for the entire Italian population. We aim to investigate the long-term risks of mortality and hospitalisation due to all causes, major diseases and cancers among Italian Balkan veterans, compared to a military cohort that had never been deployed abroad and the Italian population. The extended follow-up period, linkage with national health data and analysis of both mortality and morbidity for a wide range of causes make this epidemiological study unique, providing a more comprehensive assessment of the long-term health profile of the SEBAL cohort than previous studies.

## 2 Materials and methods

### 2.1 Data sources

The Epidemiological Observatory of the Ministry of Defense (EOD) provided personally identifiable information and deployment data for the military cohorts. Official mortality in the Italian population from 1999 to 2018 was obtained from the National Institute of Statistics (ISTAT). Causes of death are coded by ISTAT according to the International Classification of Diseases (ICD) with a standardized methodology in all regions and calendar years. Causes of death were coded with the nineth revision (ICD-9) in the period 1999 to 2002 and with the tenth revision (ICD-10) from 2003 to 2018 (29, 30).

The Ministry of Health, through a collaborative agreement with the Ministry of Defense and ISS, provided hospital discharge records (HDR) for the military cohorts from 2005 to 2018. The hospitalisation record includes the main diagnosis and up to five secondary diagnoses. All diagnoses are coded according to the nineth revision of the International Classification of Diseases – Clinical Modification (ICD-9CM) (31). Only the main diagnosis of hospitalisation was considered in the study.

The ISS Statistics Service provided data on causes of death 1999–2018 and hospitalisation 2005–2018 for the Italian population. Data were stratified by sex, calendar year, age, area of birth or of residence.

### 2.2 Study design

The study was designed as a retrospective cohort study. The exposure factor was deployment to at least one NATO peacekeeping mission in the Balkans. The date of the first mission was considered the start of exposure time for individuals with either one or multiple missions, i.e., each subject was considered at risk from the first mission onwards, regardless of the number of missions completed.

The exposed SEBAL cohort consisted of 71,144 Italian soldiers deployed in at least one of the Balkan theatres (Bosnia, Kosovo, Croatia, Albania and Macedonia) between January 1995 and December 2004.

Two unexposed control cohorts were used: the Italian male general population and a cohort of military personnel serving during the study period and not deployed abroad (Carabinieri cohort). This cohort, the same used in the first SEBAL study, included all military personnel belonging to the Carabinieri, a military police corps, who were in service on 1 January 1999 (114,260 subjects) and were never deployed abroad. Personal information of this cohort was accessible, of high quality and complete. Furthermore, the selection criteria to recruit the Carabinieri are similar to those used in other armies. Like SEBAL participants, the Carabinieri are subject to regular health surveillance during service. This shared framework helps to mitigate potential health differences at baseline.

Deaths and hospitalisations occurring in the cohorts until 2018 were the study outcomes. Only hospitalisations for acute pathological events, either ordinary or day hospital admissions, were considered. Admissions for rehabilitation, long-term hospitalisation, psychiatric diagnoses, accidental causes and poisoning, pregnancy and childbirth were excluded from the analyses as not relevant to the study.

We analyzed mortality and hospitalisation risks for the main nosological groups and for 27 main malignant tumours, either solid or hematological (Supplementary Table S1).

### 2.3 Quality checks

Data on individual personal information was checked to assess the accuracy of the information and to maximise the efficiency of linkage with mortality and hospitalisation data.

For both cohorts, we verified: (i) completeness and accuracy of personal information; (ii) internal consistency between variables within a record (e.g., between date of birth and tax code, between date of death, date of hospitalisation and foreign deployment); (iii) completeness of information on deployment to Balkan theatres (departure and return dates, destinations).

### 2.4 Record linkage

Causes of death in 1999–2018 for both cohorts were identified by deterministic record linkage with the official national mortality database. The linkage keys were the tax code and, as a secondary option, the combination of name, surname, sex, place and date of birth.

A unique linkage key based on the tax code was used for the deterministic linkage of records with the national archive of the HDR in the period 2005–2018.

### 2.5 Selection of the study cohorts

The SEBAL and Carabinieri (CC) cohorts consisted of 71,144 and 114,260 individuals, respectively. In the first dataset tax codes were missing for 8,349 (12%) subjects in the SEBAL cohort and for the whole (100%) CC cohort. Additional work to retrieve the missing information was achieved and in the final dataset the proportion of valid tax codes reached 99% for the SEBAL cohort and 100% for the CC cohort.

Quality checks of the SEBAL cohort data led to the exclusion of 102 subjects (0.14%) from the mortality analyses, due to missing or inconsistent personally identifiable information. A missing or incoherent tax code led to remove further 327 individuals from the hospitalisation analyses, resulting in the exclusion of a total of 429 individuals (0.6%) (Supplementary Table S2).

From the Carabinieri cohort, 6,002 individuals (5.25%) who were also in the SEBAL cohort and 2 subjects over 80 years of age were excluded from both the mortality and hospitalisation analyses. Additional 421 subjects (0.37%) were excluded from the hospitalisation analysis because of issues in the tax code (Supplementary Table S2).

After these exclusions the number of individuals eligible for the linkage was respectively:

- 71,042 (mortality study) and 71,015 (hospitalisation study) in the SEBAL cohort
- 108,256 (mortality study) and 107,837 (hospitalisation study) in the CC cohort.

During follow-up, a further 6,207 individuals in the CC cohort were censored because they were deployed abroad.

Among the 895 deceased subjects identified in the SEBAL cohort, no cases were found with overseas missions after their recorded date of death, confirming the reliability of deployment data. Overall, 22 deaths had inconsistencies between hospitalisation and death dates or in the personal information, but after revision, 21 deaths were confirmed (minor differences compatible with single typing errors) and only one rejected (major difference between tax code and date of birth in the ISTAT archive).

In the Carabinieri cohort 2,862 deaths were identified and 81 of them were revised. A total of 16 deaths were not confirmed due to multiple inconsistencies: two in the sequence of dates, seven in the personal data and seven in both pieces of information.

Overall, 894 and 2,846 deaths were included in the analyses for the SEBAL and CC cohorts, respectively.

### 2.6 Ethics approval

This study was performed in line with the principles of the Declaration of Helsinki. Approval was granted by the Italian Data Protection Authority (January 19, 2011/Registro dei Provvedimenti No. 017). This is an observational study. The National Ethics Committee, established at the Istituto Superiore di Sanità (ISS), has confirmed that no ethical approval is required.

### 2.7 Statistical analysis

Standardized Mortality Ratio (SMR) and Standardized Hospitalisation Ratio (SHR) were used to compare the observed risk in the SEBAL cohort with the expected risk estimated in the control populations.

For the estimation of the cause-specific risk of hospitalisation, only the first cause-specific admission was considered as an event, as it reflects the disease-specific incidence and prevents overestimation that could result from including subsequent admissions for the same cause. Conversely, the overall risk of hospitalisation included all admissions for eligible causes, i.e., multiple hospitalisations for the same cause, to reflect all diseases burden. An additional censoring cause for the Carabinieri cohort was deployment abroad to operating theatres.

To control for risk confounders, SMR estimates were standardized for age (18–29, 30–39, 40–49, 50–59, 60–69, 70–79), calendar year and area of birth (Northern/Central Italy vs. Southern Italy and Abroad). The latter is related to socioeconomic and behavioral risk factors. Area of birth abroad was assimilated to Southern Italy, which was particularly affected by migratory flows abroad in the past.

The SHR estimates were standardized for age (18–29, 30–39, 40–49, 50–59, 60–69, 70–79), calendar year and area of residence (Northern/Central Italy vs. Southern Italy), when compared to the general population, or area of birth, when compared to the Carabinieri cohort. Indeed, information on the area of birth was incomplete in the HDRs, whereas it was derived from the tax code for the military control cohort.

Mortality and hospitalisation data for the Italian male general population provided robust estimates of the expected risks across all these standardization strata, even for the rarest outcomes.

Conversely, the estimation of the expected risks in the Carabinieri cohort was critical for less common diseases, due to the much lower number of person-years at risk. Therefore, SMR and SHR were only standardized for age and area of birth when referring to the Carabinieri cohort.

When considering rare outcomes multiple controls per cases are recommended to improve the statistical power and precision of findings (32, 33). Even limiting standardization strata to age and area of birth, a minimum 2:1 ratio between control and target populations was not achieved for the younger age groups (18–29 and 30–39 years). Therefore, for adult cancers with a not negligible incidence or mortality in young age (HL, testis, skin melanoma, brain, bone, soft tissue, thyroid), SMR and SHR were estimated using only the Italian population as a reference. The age profile of incidence for these tumours differs significantly from the typical pattern of increasing incidence with age, justifying a specific weighting system in the international standardization of cancer survival by age (34).

Wald 95% confidence intervals (CIs) for SMR/SHR estimates were calculated by assuming a Poisson distribution for the observed deaths and hospitalisations (35). Analyses were performed using SAS version 9.4, STATA version 13 and R software.

## 3 Results

### 3.1 Characteristics of the study cohorts

The SEBAL cohort analyzed consisted of a maximum of 71,042 subjects, 99% of them were men (Table 1). In total, 49,369 individuals (69%) were aged 18–29 years at their first deployment to the Balkans, 20% were aged 30–39 years and 11% were over 40 years. The mean age at enrolment was 27 years (SD = 8).

**Table 1:** Characteristics of the SEBAL cohort (overall and by military corps) and of the Carabinieri control cohort.

Characteristics	SEBAL					Control cohort
	Overall, *n* = 71,042	Air, *n* = 6,544	Carabinieri, *n* = 6,275	Army, *n* = 51,687	Navy, *n* = 6,536	Carabinieri, *n* = 108,256
Age - mean (SD)	27 (8)	33 (8)	34 (7)	25 (8)	27 (6)	32 (8)
Age at enrolment^a^, *n* (%)						
18–29	49,369 (69%)	2,171 (33%)	1,822 (29%)	40,856 (79%)	4,520 (69%)	44,065 (41%)
30–39	14,552 (20%)	2,957 (45%)	3,144 (50%)	6,661 (13%)	1,790 (27%)	47,781 (44%)
40–49	6,236 (8.8%)	1,214 (19%)	1,243 (20%)	3,560 (6.9%)	219 (3.4%)	13,530 (12%)
50–59	879 (1.2%)	202 (3.1%)	66 (1.1%)	604 (1.2%)	7 (0.1%)	2,842 (2.6%)
60–69	6 (<0.1%)	0 (0%)	0 (0%)	6 (<0.1%)	0 (0%)	32 (<0.1%)
70–79						6 (<0.1%)
Sex, *n* (%)						
Female	432 (0.6%)	6 (<0.1%)	100 (1.6%)	325 (0.6%)	1 (<0.1%)	2 (<0.1%)
Male	70,610 (99%)	6,538 (100%)	6,175 (98%)	51,362 (99%)	6,535 (100%)	108,254 (100%)
Area of birth, *n* (%)						
Italy, North (N)	8,387 (12%)	480 (7.3%)	1,284 (20%)	6,063 (12%)	560 (8.6%)	19,939 (18%)
Italy, Centre (C)	10,105 (14%)	1,499 (23%)	1,108 (18%)	6,884 (13%)	614 (9.4%)	19,032 (18%)
Italy, South (S)	35,213 (50%)	3,484 (53%)	2,406 (38%)	25,062 (48%)	4,261 (65%)	44,308 (41%)
Italy, Islands (I)	14,356 (20%)	825 (13%)	1,144 (18%)	11,561 (22%)	826 (13%)	22,245 (21%)
Abroad (A)	2,981 (4.2%)	256 (3.9%)	333 (5.3%)	2,117 (4.1%)	275 (4.2%)	2,732 (2.5%)
Year of first mission, *n* (%)						
1995	2,157 (3.0%)	0 (0%)	7 (0.1%)	287 (0.6%)	1,863 (29%)	
1996	4,360 (6.1%)	0 (0%)	394 (6.3%)	3,843 (7.4%)	123 (1.9%)	
1997	4,259 (6.0%)	1 (<0.1%)	219 (3.5%)	3,835 (7.4%)	204 (3.1%)	
1998	3,826 (5.4%)	16 (0.2%)	426 (6.8%)	2,918 (5.6%)	466 (7.1%)	
1999	13,681 (19%)	60 (0.9%)	1,132 (18%)	10,149 (20%)	2,340 (36%)	
2000	13,253 (19%)	1,805 (28%)	1,109 (18%)	9,506 (18%)	833 (13%)	
2001	10,834 (15%)	1,691 (26%)	419 (6.7%)	8,553 (17%)	171 (2.6%)	
2002	8,815 (12%)	1,565 (24%)	598 (9.5%)	6,456 (12%)	196 (3.0%)	
2003	5,662 (8.0%)	952 (15%)	1,022 (16%)	3,482 (6.7%)	206 (3.2%)	
2004	4,195 (5.9%)	454 (6.9%)	949 (15%)	2,658 (5.1%)	134 (2.1%)	
Number of missions, *n* (%)						
1	26,314 (37%)	3,675 (56%)	3,529 (56%)	16,608 (32%)	2,502 (38%)	
2	14,198 (20%)	1,717 (26%)	1,195 (19%)	9,942 (19%)	1,344 (21%)	
3	10,618 (15%)	708 (11%)	579 (9%)	8,329 (16%)	1,002 (15%)	
4	7,851 (11%)	281 (4%)	354 (6%)	6,604 (13%)	612 (9%)	
5	5,181 (7%)	101 (2%)	236 (4%)	4,509 (9%)	335 (5%)	
>5	6,880 (10%)	62 (1%)	382 (6%)	5,695 (11%)	741 (11%)	

^a^Age at first deployment to the Balkans for the SEBAL cohort and at 1/1/1999 for the Carabinieri control cohort. Numbers and proportions (%).

The Carabinieri control cohort included 108,256 subjects, all of whom were men but two women. On 1/1/1999 most subjects were aged 30–39 years (47,781; 44%) and 18–29 years (44,065; 41%), with a mean age of 32 years (SD = 8).

Most subjects were born in Southern Italy or abroad: 52,550 (74%) in the SEBAL cohort (70% in Southern regions and Islands and 4% abroad) and 69,285 (64%) in the Carabinieri cohort (only 2.5% abroad), respectively. The vast majority of the SEBAL cohort belonged to the Army (51,687 subjects), whereas a minority, equally distributed, came from the Air Force, Carabinieri and Navy corps.

The majority of Balkan veterans served in more than one mission (44,728; 63%) and 26,314 subjects (37%) participated in one mission only. The proportion of individuals with single missions was higher among Air Force and Carabinieri personnel (56%).

During the mortality follow-up period the total number of person-years at risk in the SEBAL cohort was 1,309,162 (Supplementary Table S3a). The number of person-years increased gradually from 1999 to 2004, in line with the first deployment to the Balkans. Over the same period the number of person-years in the CC cohort was higher (2,091,738) and changed over time due to death or censoring for deployment abroad. The ratio of person-years at risk in the CC cohort to the SEBAL cohorts was 1.6 overall and only above 2:1 for the 40–49 years and older age groups. For the younger age groups, instead, the CC/SEBAL ratio was on average 0.7 (18–29 years) and 1.3 (30–39 years) and fell well below 1:1 with time, particularly for subjects born in the South, who were the majority (Supplementary Figure S1).

The number of person-years at risk of hospitalisation in the CC (1,423,967) and SEBAL (983,217) cohorts resulted in an overall ratio of 1.4, which decreased further for the younger age groups (Supplementary Table S3b). The overall CC/SEBAL ratio was 0.4 for the 18–29 year age group, corresponding to less than 1 control for every 2 cases, and it decreased over time, approaching zero in 2011.

### 3.2 Mortality and hospitalisation results

The risk of hospitalisation and death from all causes and all natural causes of disease in the SEBAL cohort was lower than expected, compared to both control populations.

**Figure 1:**
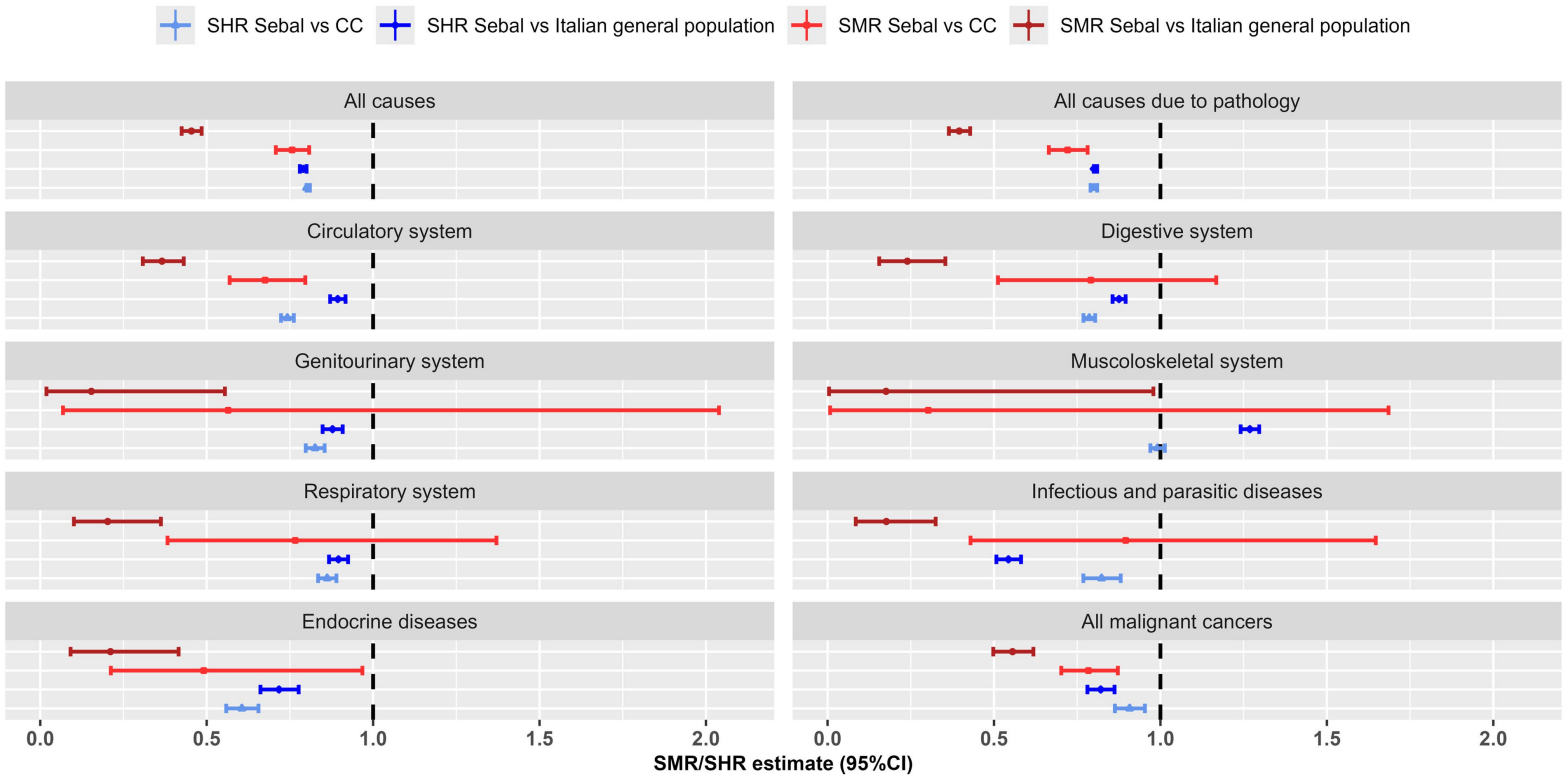
Major nosological groups: Standardized Mortality Ratio (SMR) and Standardized Hospitalisation Ratio (SHR), with 95% confidence interval (95% CI), for the SEBAL cohort using the Italian population and the Carabinieri (CC) control cohort as reference. Mortality data 1999–2018, hospitalisation data 2005–2018.

In the SEBAL cohort the observed number of deaths from *all causes* was 894 against 1,969 expected in a comparable group from the general population (SMR = 0.45; 95%CI: 0.42–0.48) and 1,182 from the CC cohort (SMR = 0.76; 95%CI: 0.71–0.81) (Table 2; Figure 1). The observed total number of hospital admissions for all causes (including multiple admissions for the same pathology in the same individual) was 68,712 against 86,949 and 85,688 expected, respectively, in the general population (SHR = 0.79; 95%CI: 0.78–0.80) and in the CC cohort (SHR = 0.80; 95%CI 0.80–0.81). The crude all causes mortality rate in the SEBAL cohort was 68 per 100,000 and the standardized rate was 309 per 100,000, using the general population as a reference (Supplementary Table S4).

**Table 2:** Mortality 1999–2018 and hospitalisation 2005–2018 in the SEBAL cohort for the main nosological groups.

Cause of disease	Observed events, SEBAL cohort		Expected mortality, SEBAL cohort								Expected hospitalisation, SEBAL cohort							
			Carabinieri ref.				Italian population ref.				Carabinieri ref.				Italian population ref.			
	Deaths	Hospitalisations	Expected deaths	SMR	95% CI		Expected deaths	SMR	95% CI		Expected hosp.	SHR	95%CI		Expected hosp.	SHR	95% CI	
All causes^a^	894	68,712	1,182	**0.76**	0.71	0.81	1,969	**0.45**	0.42	0.48	85,688	**0.80**	0.80	0.81	86,949	**0.79**	0.78	0.80
All causes due to pathology^a^	602	60,491	835	**0.72**	0.66	0.78	1,522	**0.40**	0.36	0.43	75,678	**0.80**	0.79	0.81	75,149	**0.80**	0.80	0.81
Circulatory system	142	5,751	210	**0.68**	0.57	0.80	388	**0.37**	0.31	0.43	7,747	**0.74**	0.72	0.76	6,435	**0.89**	0.87	0.92
Digestive system	25	7,728	32	0.79	0.51	1.17	104	**0.24**	0.16	0.35	9,830	**0.79**	0.77	0.80	8,825	**0.88**	0.86	0.90
Genitourinary system	2	3,283	4	0.56	0.07	2.04	13	**0.15**	0.02	0.55	3,976	**0.83**	0.80	0.85	3,739	**0.88**	0.85	0.91
Muscoloskeletal system	1	7,986	3	0.30	0.01	1.69	6	**0.18**	0.00	0.98	8,056	0.99	0.97	1.01	6,295	**1.27**	1.24	1.30
Respiratory system	11	3,797	14	0.77	0.38	1.37	54	**0.20**	0.10	0.36	4,405	**0.86**	0.83	0.89	4,238	**0.90**	0.87	0.92
Infectious and parasitic diseases	10	842	11	0.90	0.43	1.65	57	**0.18**	0.08	0.32	1,023	**0.82**	0.77	0.88	1,550	**0.54**	0.51	0.58
Endocrine diseases	8	613	16	**0.49**	0.21	0.97	38	**0.21**	0.09	0.42	1,012	**0.61**	0.56	0.66	855	**0.72**	0.66	0.78
All cancers	336	1,579	429	**0.78**	0.70	0.87	605	**0.56**	0.50	0.62	1,740	**0.91**	0.86	0.95	1,924	**0.82**	0.78	0.86

^a^The number of hospitalisations includes multiple discharges for each subject. Number of observed and expected events based on the risk profile of the Italian population and of the Carabinieri control cohort as reference (ref.). Standardized Mortality Ratio (SMR) and Standardized Hospitalisation Ratio (SHR) with 95% confidence interval (95%CI). Statistically significant values in bold.

For *all natural causes of disease*, the SEBAL cohort had a mortality risk that was more than halved (SMR = 0.40, 0.36–0.43) compared to the general population and a hospitalisation risk that was 20% lower (SHR = 0.80; 0.80–0.81). The health status of the SEBAL cohort was also more favorable than that of the Carabinieri control group. For all natural causes the risk of death was 28% lower (SMR = 0.72; 0.66–0.78) and the risk of hospitalisation was 20% lower than expected (SHR = 0.80, 0.79–0.81) (Table 2; Figure 1). The crude mortality rate for all natural causes in the SEBAL cohort was 46 per 100,000 and the standardized rate was 253 per 100,000, using the general population as a reference (Supplementary Table S4).

#### 3.2.1 Major nosological groups

For almost all major groups of diseases the SEBAL cohort showed lower than expected risk of hospitalisation and mortality compared to the general population. The most relevant decreases were observed for infectious diseases (SHR = 0.54, SMR = 0.18) and for diseases of the endocrine system (SHR = 0.72, SMR = 0.21). Decreased risks were also observed for diseases of the circulatory system (SHR = 0.89; 0.87–0.92, SMR = 0.37; 0.31–0.43), digestive system (SHR = 0.88; 0.86–0.90, SMR = 0.24; 0.16–0.35), genitourinary (SHR = 0.88; 0.85–0.91, SMR = 0.15; 0.02–0.55), respiratory (SHR = 0.90; 0.87–0.92, SMR = 0.20; 0.10–0.36) and for all malignant tumors (Table 2; Figure 1).

For all above mentioned diseases the hospitalisation risk was reduced even in comparison to the CC control cohort. Conversely the mortality risk did not differ significantly for diseases of digestive, genitourinary and respiratory system and for infectious diseases, being lower only for diseases of circulatory or endocrine system and for all cancers (Table 2; Figure 1).

Hospitalisations for diseases of the musculoskeletal system were the only exception to this scenario. The hospitalisation risk in the SEBAL cohort was 27% higher compared to the general population (SHR = 1.27; 1.24–1.30) and similar to that of the Carabinieri cohort (SHR = 0.99; 0.97–1.01). Coherently, the Carabinieri control population had a higher hospitalisation risk for musculoskeletal injuries than the Italian population (SHR = 1.16; 1.14–1.19; data not shown).

#### 3.2.2 Cancers

**Figure 2:**
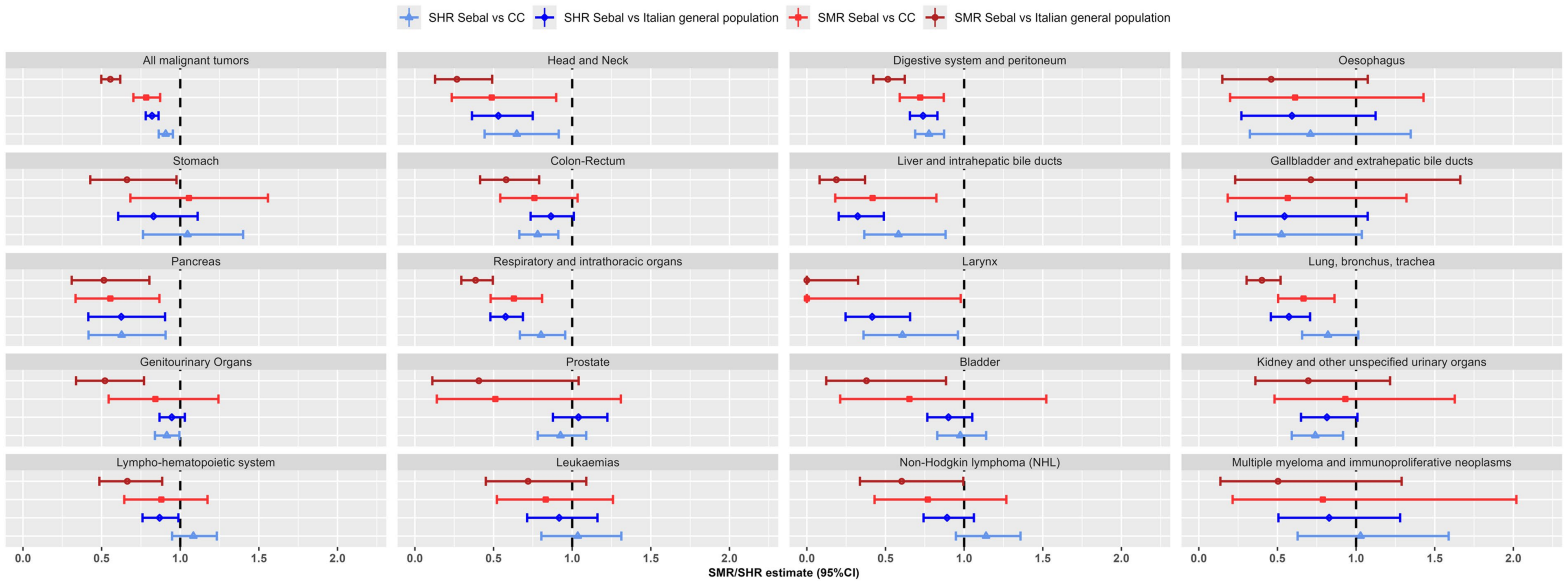
Most frequent cancers: Standardized Mortality Ratio (SMR) and Standardized Hospitalisation Ratio (SHR), with 95% confidence interval (95% CI), for the SEBAL cohort using the Italian population and the Carabinieri (CC) control cohort as reference. Mortality data 1999–2018, hospitalisation data 2005–2018.

For *all malignant tumours* combined, we identified 336 deaths and 1,579 individuals with at least one hospital discharge in the SEBAL cohort. Cancer hospitalisation risk was lower than expected based on observation of both the general population (SHR = 0.82, 0.78–0.86) and the Carabinieri cohort (SHR = 0.91, 0.86–0.95). Risk of cancer death was further reduced: by 44% versus the general population (SMR = 0.56, 0.50–0.62) and by 22% versus the Carabinieri (SMR = 0.78, 0.70–0.87) (Table 3; Figure 2).

**Table 3:** Mortality 1999–2018 and hospitalisation 2005–2018 in the SEBAL cohort for the most frequent cancers.

Cancers	Observed events, SEBAL cohort		Expected Mortality, SEBAL cohort								Expected Hospitalisation, SEBAL cohort							
			Carabinieri ref.				Italian population ref.				Carabinieri ref.				Italian population ref.			
	Deaths	Hospitalisations	expected deaths	SMR	95% CI		Expected deaths	SMR	95% CI		expected hosp.	SHR	95% CI		expected hosp.	SHR	95% CI	
All cancers^a^	336	1,579	429	**0.78**	0.70	0.87	605	**0.56**	0.50	0.62	1,740	**0.91**	0.86	0.95	1,924	**0.82**	0.78	0.86
Head and Neck	10	32	20	**0.49**	0.23	0.90	37	**0.27**	0.13	0.49	49	**0.65**	0.44	0.91	60	**0.53**	0.36	0.75
Digestive system and peritoneum	107	283	148	**0.72**	0.59	0.87	208	**0.52**	0.42	0.62	364	**0.78**	0.69	0.87	383	**0.74**	0.66	0.83
Oesophagus	5	9	8	0.61	0.20	1.43	11	0.46	0.15	1.07	13	0.71	0.32	1.35	15	0.59	0.27	1.12
Stomach	25	45	24	1.06	0.68	1.56	38	**0.66**	0.43	0.98	43	1.05	0.76	1.40	54	0.83	0.61	1.11
Colon-rectum	40	159	53	0.76	0.54	1.03	69	**0.58**	0.41	0.79	204	**0.78**	0.66	0.91	184	0.86	0.74	1.01
Liver and intrahepatic bile ducts	8	22	19	**0.42**	0.18	0.82	43	**0.19**	0.08	0.37	38	**0.58**	0.37	0.88	68	**0.32**	0.20	0.49
Gallbladder and extrahepatic bile ducts	5	8	9	0.57	0.18	1.32	7	0.71	0.23	1.66	15	0.53	0.23	1.04	15	0.54	0.24	1.07
Pancreas	19	28	34	**0.56**	0.33	0.87	37	**0.51**	0.31	0.80	45	**0.63**	0.42	0.91	45	**0.63**	0.42	0.90
Respiratory and intrathoracic organs	61	125	97	**0.63**	0.48	0.81	158	**0.39**	0.30	0.50	156	**0.80**	0.67	0.96	217	**0.58**	0.48	0.69
Larynx	0	18	4	0.00	0.00	0.98	11	0.00	0.00	0.33	30	**0.61**	0.36	0.96	43	**0.42**	0.25	0.66
Lung, bronchus, trachea	57	86	86	**0.67**	0.50	0.86	142	**0.40**	0.30	0.52	105	0.82	0.66	1.01	150	**0.57**	0.46	0.71
Genitourinary organs	25	542	30	**0.84**	0.54	1.24	48	**0.52**	0.34	0.77	593	**0.91**	0.84	0.99	573	0.95	0.87	1.03
Prostate	4	145	8	0.51	0.14	1.31	10	0.41	0.11	1.04	157	0.93	0.78	1.09	139	1.04	0.88	1.22
Bladder	5	159	8	0.65	0.21	1.52	13	**0.38**	0.12	0.88	164	0.97	0.82	1.13	177	0.90	0.77	1.05
Kidney and other unspecified urinary organs	12	84	13	0.93	0.48	1.63	17	0.70	0.36	1.22	113	**0.74**	0.59	0.92	103	0.81	0.65	1.01
Lympho-hematopoietic system	46	231	52	**0.88**	0.64	1.17	69	**0.66**	0.49	0.89	213	1.08	0.95	1.23	266	**0.87**	0.76	0.99
Leukaemias	22	69	26	0.83	0.52	1.26	31	0.72	0.45	1.09	67	1.03	0.80	1.30	75	0.92	0.71	1.16
Non-Hodgkin lymphoma (NHL)	15	125	20	0.77	0.43	1.27	25	**0.60**	0.34	0.99	110	1.14	0.95	1.36	140	0.89	0.74	1.06
Multiple myeloma and immunoproliferative neoplasms	4	20	5	0.79	0.21	2.02	8	0.50	0.14	1.29	19	1.03	0.63	1.59	24	0.83	0.51	1.28

^a^Excluding malignant skin tumours other than melanomas. Number of observed and expected events based on the risk profile of the Italian population and of the Carabinieri control cohort as reference (ref.). Standardized Mortality Ratio (SMR) and Standardized Hospitalisation Ratio (SHR) with 95% confidence interval (95%CI). Statistically significant values in bold.

As for all *solid cancers*, the risk of hospitalisation and death in the SEBAL cohort was reduced, compared to both control cohorts, for *head and neck* cancers (SHR = 0.53 and SMR = 0.27 vs. general population), for cancers of the *digestive system* (SHR = 0.74 and SMR = 0.52), the *respiratory system* (SHR = 0.58 and SMR = 0.39), and of liver, pancreas, lung and larynx. Lower, or not significantly different, risk was found for cancers of the oesophagus, stomach, colon-rectum, gallbladder and bile ducts, prostate, bladder and kidney and in general for genitourinary system tumours (SHR = 0.92; 0.87–1.03 and SMR = 0.52; 0.34–0.77), (Table 3; Figure 2).

For *all haematolymphoid neoplasms*, 46 deceased and 231 discharged subjects were identified in the SEBAL cohort. Hospitalisation and death risks were lower than those expected in the Italian population (SHR = 0.87; 0.76–0.99 and SMR = 0.66; 0.49–0.89) and not significantly different than in the Carabinieri cohort (SHR = 1.08; 0.95–1.23; SMR = 0.88; 0.64–1.17).

Similarly, specific haematolymphopoietic neoplasms did not show any statistically significant increase in risk for the military personnel deployed in the Balkans. For leukemia, the first cause of death from hematological neoplasms in the SEBAL cohort (22 out of 46 total deaths), mortality risk was lower than in the general population, but the difference was not statistically significant (SMR = 0.72; 0.45–1.09). For non-Hodgkin lymphomas (NHL), the most frequent diagnosis of admission (125 out of 231 individuals discharged), the hospitalisation risk was lower and not significantly different than that expected in the Italian population (SHR = 0.89; 0.74–1.06). Compared to the Carabinieri cohort, the risk of hospitalisation for NHL, leukemia or multiple myeloma, was approximately 3–14% higher than expected, while the corresponding risk of death was 17–23% lower. However, none of these differences were statistically significant at the 95% confidence level (Table 3; Figure 2).

#### 3.2.3 Cancers in young adults

Given the young median age at the date of deployment, particular attention was paid to tumours affecting the young adult male population. In the SEBAL cohort juvenile cancers were the cause of death in 75 subjects (22% of the 336 total cancer deaths) and the main diagnosis of hospitalisation for 538 subjects (34% of the 1,579 total cancer hospitalisations) (Table 4).

**Table 4:** Mortality 1999–2018 and hospitalisation 2005–2018 in the SEBAL cohort for the most frequent cancers in young adults.

Cancers	Observed events, SEBAL cohort		Expected mortality, SEBAL cohort				Expected hospitalisation, SEBAL cohort			
	Deaths	Hospitalisations	Expected deaths	SMR	95% CI		Expected hosp.	SHR	95% CI	
Bone and joint cartilages	2	11	6	0.33	0.04	1.20	19	0.59	0.29	1.05
Connective and soft tissue	8	34	8	0.97	0.42	1.92	33	1.02	0.71	1.42
Skin melanoma	19	94	17	1.13	0.68	1.76	71	**1.32**	1.07	1.61
Testis	4	158	6	0.62	0.17	1.59	162	0.98	0.83	1.14
Central Nervous System (CNS)	35	66	38	0.91	0.64	1.27	79	0.84	0.65	1.07
Brain	34	60	38	0.90	0.63	1.26	73	0.82	0.63	1.06
Thyroid	2	142	2	1.01	0.12	3.66	102	**1.39**	1.17	1.64
Hodgkin lymphoma	5	33	6	0.84	0.27	1.97	53	**0.62**	0.43	0.87

Number of observed and expected events based on the risk profile of the Italian population as reference. Standardized Mortality Ratio (SMR) and Standardized Hospitalisation Ratio (SHR) with 95% confidence interval (95% CI). Statistically significant values in bold.

Central nervous system (CNS) tumours were the leading cause of death, accounting for 35 of the 75 overall deaths (47%), whereas testicular and thyroid cancers were the most common diagnoses for hospitalisation (158 and 142 observed events, respectively).

**Figure 3:**
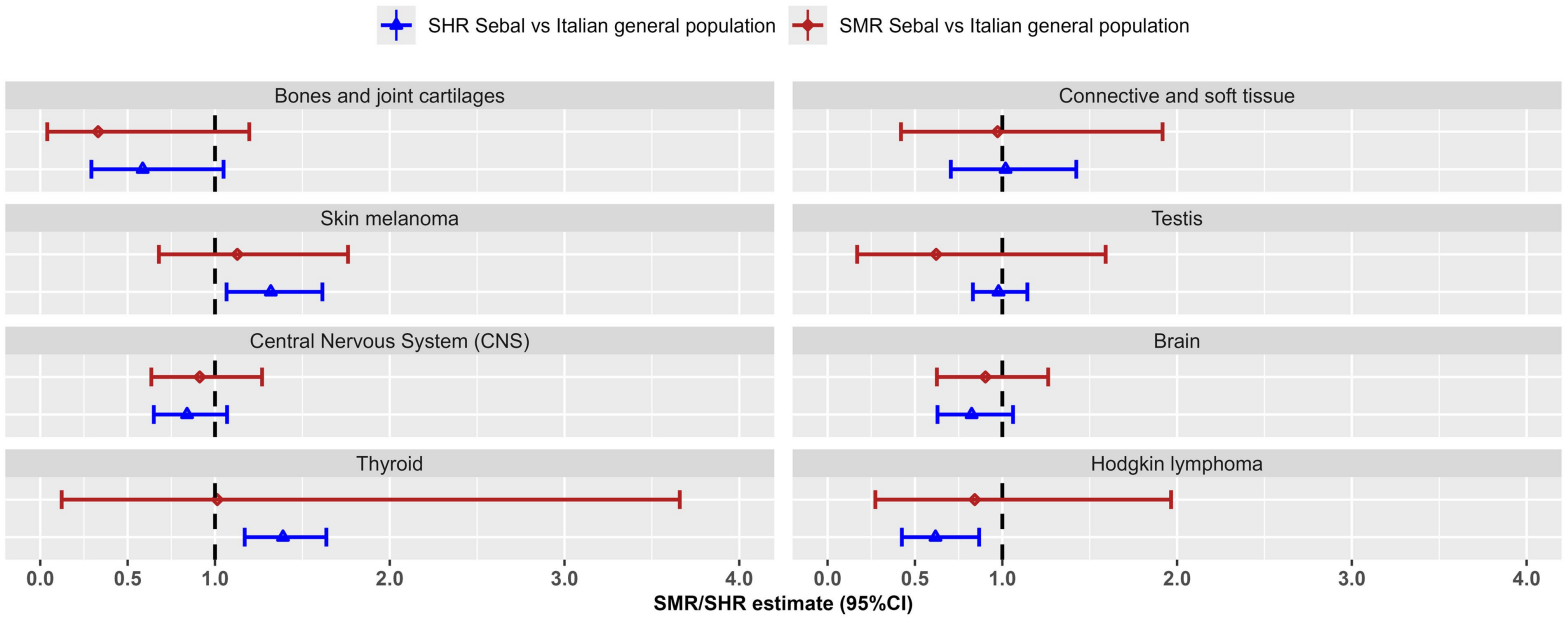
Most frequent cancers in young adults: Standardized Mortality Ratio (SMR) and Standardized Hospitalisation Ratio (SHR), with 95% confidence interval (95% CI), for the SEBAL cohort using the Italian population as reference. Mortality data 1999–2018, hospitalisation data 2005–2018.

Compared to the Italian population, no statistically significant differences from expectation were estimated for cancers of bone, soft tissue, testis and CNS. For these neoplasms, the risk of death or hospitalisation was lower (or at most equal), but the differences from the expected value never reached the statistical significance (Table 4; Figure 3).

The risk of hospitalisation for Hodgkin lymphoma was 38% lower than expected in the general population (SHR = 0.62; 0.43–0.87). A parallel 16% reduction in the mortality risk was estimated (SMR = 0.84), but this reduction, based on 5 observed deaths, did not reach the statistical significance (95%CI 0.27–1.97).

For cutaneous melanoma (94 hospitalisations and 19 deaths) and thyroid cancer (142 hospitalisations and 2 deaths), we estimated an increased risk of hospitalisation in the SEBAL cohort compared to the general population (SHR = 1.32; 1.07–1.61 and SHR = 1.39; 1.17–1.64, respectively). This increased risk of hospitalisation is partly reflected in the slightly higher, but not significantly different, risk of death from skin melanoma (SMR = 1.13; 0.68–1.76) and thyroid cancer (SMR = 1.01; 0.12–3.66), Table 4; Figure 3.

### 3.3 Sensitivity analysis

We performed a sensitivity analysis on the impact of multiple deployments to the Balkans, stratifying the computation of the SHR with respect to the number of missions (single vs. multiple). Participants in single missions generally had a less favorable SHR than those with multiple missions. For example, the SHR for all natural causes was 0.86 (95% CI 0.85–0.87) and 0.77 (95% CI 0.76–0.78) for individuals with single and multiple missions, respectively. However, no additional excess risks were observed in the two subgroups beyond those observed in the overall group, for either major diseases or neoplasms (Supplementary Tables S5, S6).

## 4 Discussion

The study findings indicate that the overall health profile of the SEBAL cohort was more favorable compared to both control populations: the Italian general population and a cohort of military personnel never deployed abroad (Carabinieri cohort). The reduced mortality risk for *all causes* in comparison to the general population is in line with what observed in the first SEBAL study (28) and in similar studies performed in Europe (20, 22, 36, 37). The most recent of these studies reports a comparable value for the pool of Nordic military veterans following international deployment (SMR = 0.58; 0.52–0.64) (22). Our findings showed a lower mortality risk than that reported in a meta-analysis of 59 international studies, which showed a maximum 25% reduction in all-cause mortality for military personnel (38).

As for *disease-related causes*, the health profile of the Balkan veterans was similar to that of an active professional population. This confirms the so-called “healthy soldier” effect, a term borrowed from the professional context (healthy worker effect), to indicate that military personnel are a selected population, based on good health conditions and physical performance (39).

More specifically, for all main groups of pathologies, but musculoskeletal system, we found a reduced risk of hospitalisation in the SEBAL cohort compared to the general population and an even lower risk of death. Interestingly, *for infectious diseases* the relative risk of hospitalisation and death were both very low. This result suggests that better vaccine coverage of military personnel may play a role, although this hypothesis was not specifically verified. These results are consistent with a retrospective study on Italian Gulf War veterans (40).

The hospitalisation risk for *musculoskeletal system injuries* was similar to the Carabinieri cohort, who also experienced a higher risk than the general population (SHR = 1.16, 1.14–1.19, data not shown). An excess in the hospitalisation risk of musculoskeletal pathologies was also observed in Italian Gulf War veterans (40). Musculoskeletal system pathologies, especially meniscal and cruciate ligament injuries of the knee, are reported as frequent diagnoses for hospitalisation in active-duty military personnel (41, 42). These injuries are more common with increasing age and in men. Reasons are attributable to the peculiarities of the physical activity performed during operational military service. The impact on mortality, as expected, was negligible.

The study focused on assessing the risk of mortality and hospitalisation due to cancer, a cause of concern due to the potential exposure to diverse carcinogenic factors during deployment to the Balkans. Our findings indicate a reduced risk of death and hospitalisation for *malignant tumors* in the SEBAL cohort compared to both control populations. These results are coherent with other European studies investigating cancer occurrence in veterans who served in peacekeeping missions in the Balkans. A lower cancer incidence risk was also found in the study on Dutch veterans (19), while no significant excess in cancer incidence was found in the veterans from other countries (17, 18, 20, 36). The previous SEBAL study estimated no significant differences in cancer mortality with the Carabinieri cohort (28). The additional 10 years of follow-up enabled more precise estimates. The small, but significant, risk difference between the two military cohorts could be related to individual risk factors, such as smoking habits. Indeed, the mortality risk for smoking-related cancers was significantly lower in the SEBAL cohort than in the Carabinieri control group.

A modest but significant excess risk of hospitalisation in the SEBAL cohort over the Italian population was estimated for thyroid cancer and cutaneous melanoma, with no significant effect on mortality. The Carabinieri cohort also exhibits an increased risk of hospitalisation for thyroid cancer (SHR = 1.46; 1.29–1.65, data not shown). An excess incidence of thyroid cancer in the military population was also highlighted by the studies based on the Ministry of Defense’s surveillance system (21, 24, 25). These findings may reflect a greater surveillance intensity. The military population undergoes more systematic medical check-ups than the general population and this increases the risk of overdiagnosis, which is well-documented for thyroid cancer. An increase in diagnostic intensity results in the detection of subclinical and asymptomatic tumours (particularly small papillary carcinomas). This renders thyroid tumours one of the neoplasms at greatest risk of overdiagnosis and inappropriate care, particularly in instances where active population surveillance is instituted (43, 44).

Ultraviolet radiation is the leading risk factor for the development of cutaneous melanoma (cM). Individual biological factors, such as phototype and some genetic and hereditary factors compromising DNA repair mechanisms, further modulate this risk (45). Data on these risk factors were not available for the studied military cohorts. An increased risk of cM has been also repeatedly reported for active duty US Military personnel (46–48). Similarly, the increased incidence of skin melanoma could be due to greater sun exposure associated with deployment’s occupational duties. Furthermore, as with thyroid cancer, an association between increased diagnostic intensity and overdiagnosis has been identified for cM (49). Indeed, the incidence of cM is rising sharply in many countries (including Italy), whereas mortality is only slightly so and is even declining in the 20–44 age group (50). A similar discrepancy between incidence (in excess) and mortality (not in excess) for both thyroid cancer and cutaneous melanoma is shown in our study.

For *all other solid cancers*, the risk of hospitalisation and death was estimated to be lower or not significantly different. To be noted the consistency of the results for *smoking-related cancers* (lung, larynx, head and neck). The Balkan veterans showed a reduced risk of hospitalisation and death for all these cancers compared with both reference populations.

The risks of death and hospitalisation for *hematological neoplasms* were found to be lower than in the general population. Mortality results are consistent with the former SEBAL study (28). No significant differences were found with respect to the Carabinieri cohort. Coherently, the risk of death and hospitalisation for hematological neoplasms in the Carabinieri cohort was lower than in the general population (SMR = 0.76, 0.64–0.91; SHR = 0.87, 0.79–0.95, data not shown).

*Hodgkin lymphoma* (HL), which has prompted the initiation of the health surveillance among Balkan veterans, deserves special attention. HL is a relatively rare neoplasm (incidence is about 3–5 per 100,000) which comprises different subtypes (51, 52). In adults, the risk of HL occurrence has two peaks, in youth (15–30 years) and in old age (over 65 years). The etiology of HL is only partially understood. Risk factors associated with HL with sufficient scientific evidence include HIV and Epstein–Barr virus (EBV) infection (53).

It is noteworthy that 4 of the 5 total observed deaths for HL were concentrated in the period 2003–2005 and in the youngest age group (<29 years). This concentration reflects the evolution of the age structure of the SEBAL cohort. In fact, the peak of deaths occurs precisely in the years when the age group <29 years was the most numerous and the cohort was most at risk of developing HL. This result is also consistent with the study showing an isolated peak in HL incidence in the early 2000s for the Balkan veterans (25).

### 4.1 Limitations

We acknowledge some limitations in our study. First, the inability to directly examine the incidence of cancer in the Balkan veterans. In Italy, the lack of a national cancer registry impairs to obtain complete results from individual linkage with registries data. The only individual health flows available for the entire national population are those used in our study, namely the causes of death and the hospital discharges.

Secondly, the follow-up period for the study of hospitalisations (2005–2018) was more limited than that available for mortality (1999–2018). This limitation is due to the unavailability of data and cannot be overcome. Data on HDR were digitized since 2000 and over 2000–2004 lacked sufficient territorial coverage and quality to guarantee acceptable completeness. However, veterans who were hospitalised and died in the period 1999–2004 were traced in the mortality data.

Hospitalisation data from 2005 to 2018 may lead to an underestimation of conditions with short latency after deployment between 1995 and 2004. This limitation suggests that some less severe or quickly emerging conditions might have been missed.

A further limitation is that observed admissions may refer to individuals who had already been admitted for the same diagnosis prior to 2005 and before their deployment. Such hospitalisations should be excluded because not related to the Balkan mission, however, they are not identifiable. Failure to exclude these admissions, and not considering a latency period between the first mission and the occurrence of the event, may result in an overestimation of the hospitalisation risk but will never result in an underestimation.

We only considered the primary diagnoses of hospitalisation and this may have limited the identification of all morbid events. We could not utilise alternative selection algorithms because information on dates of diagnosis was not available for diseases reported among secondary diagnoses. However, risk comparisons were not compromised because all events were identified through the primary diagnosis of hospitalisation, either in the SEBAL cohort or in the two control populations.

Estimating the expected risks in the Carabinieri cohort was more challenging for rare diseases and may be affected by sparse data. Additionally, when considering multiple comparisons for a wide range of diseases, casual findings cannot be ruled out.

The SEBAL cohort included veterans who had been deployed to the Balkan theatre one or more times, which may have introduced a survival selection bias. The results of the sensitivity analysis suggest a mild survival bias, but confirmed that it did not affect the validity of the overall results.

Our study shares with similar international studies the lack of detailed information on individual exposures and lifestyle factors, which precludes the ability to draw direct causal relationships between deployment in the Balkans and health outcomes. Matching with comparators could only be done using demographic variables (e.g., sex, age and geographic origin). Alternative designs adjusting for individual level confounders such as health status or lifestyle (37, 54, 55) were not possible. Unmeasured lifestyle factors may differ between deployed and non-deployed personnel, which could influence the outcomes. For instance, the lowest SMR and SHR for all causes among the veterans compared to the CC could be related to better health conditions at baseline and to different smoking habits, as suggested by the consistent differences found for smoke-related cancers.

No direct or indirect measures of exposition to DU or other environmental pollutants were available for the Balkan veterans in Italy or in other countries where similar retrospective studies were conducted (7–22). However, none of these studies, included ours, provided evidence that exposure to DU could be relevant for the military personnel involved.

Studies conducted on a sample of nearly 1,000 Italian veterans of the Second Gulf War found no evidence of exposure to DU in the operational theatre (56), nor any correlation between long-term cancer hospitalisation and pre-post deployment concentrations of DU and other genotoxic xenobiotics (40).

Trauma or psychiatric conditions can be related to deployment conditions, and the impact of these conditions on veterans has been the subject of investigation (37, 54, 55). Although these health outcomes deserve careful consideration, they were not included among the possible causes of disease and remain to be addressed in future studies.

### 4.2 Strenghts

Methodologically, the study exhibits multiple strengths, both in its design and dimension.

To our knowledge this is the first study assessing the health status of the Balkan veterans by integrating information on both mortality and morbidity. This enabled a more accurate assessment of the risk of cancers with a favorable prognosis, for which mortality is an inadequate outcome indicator. Furthermore, the comparison of the two indicators facilitated both the validation and the interpretation of the observed results.

The mortality estimates for the SEBAL cohort are based on more than 1.3 million person-years, which is more than double those considered in the first SEBAL study, and the estimates of hospitalisation refer to nearly 1 million person-years. The corresponding values for the Carabinieri cohort are even larger, at 2.1 million for mortality and 1.4 million for hospitalisation. These dimensions lend undoubted robustness to the study and have also made it possible to estimate rare cancers with incidence rates around units per million.

Cross-referencing with national archives of causes of death and, for the first time, hospital admissions allowed a comprehensive assessment of the health status of the Balkan cohort, regardless of service status (active or discharged) and place of residence and/or hospitalisation. This feature makes the study more comprehensive than previous studies based on spontaneous reporting of cancer cases among military personnel (21, 23–25). These were inevitably limited by under-reporting of military personnel on leave from active service and by the comparison with local control populations (26, 27).

Another methodological advantage of the study design is the homogeneous quality of the data on causes of death and hospital diagnoses. Indeed, uniform and comparable coding procedures and data quality checks, by region and over time, are applied centrally to both mortality and HDR archives.

A double reference population was used in the study. The Italian population is the natural reference to have robust control values, even for rare diseases. The Carabinieri cohort, the same used in the first SEBAL study (28), was confirmed to be a control group with better health conditions than the general male population. The double comparison provided useful elements for interpretation, for example in assessing the excess risks found for musculoskeletal injuries or for cancers on which active surveillance has a greater impact.

## 5 Conclusion

The study confirms and extends the findings of the previous retrospective mortality study, thanks to 10 years longer follow-up and simultaneous analysis of hospitalisation and mortality risks. The results of this long-term observational study do not support the hypothesis that military service in peacekeeping missions in the Balkans is associated with an increased risk of mortality or hospitalisation from natural causes, including malignant tumours.

Our findings are consistent with a healthy soldier effect and do not indicate any additional long-term health risks attributable to deployment.

The methodological approach followed in our study offers insights for the establishment of a health surveillance system for military personnel, based on systematic cross-referencing with national health records. The methodology employed in this study can also be applied in other contexts where the population is not fully covered by disease registers or is covered for an insufficient duration.

## Data Availability

The datasets presented in this article are not readily available because of their sensitive nature. Requests to access the datasets should be directed to silvia.rossi@iss.it.
