# Supplementary material for: Long-term mortality and hospitalisation among Italian veterans deployed to the Balkans: a retrospective longitudinal study based on individual linkage with national health data
Source: Front Public Health. 2025 Sep 19;13:1605457. doi: 10.3389/fpubh.2025.1605457 (PMC12491181; doi:10.3389/fpubh.2025.1605457)
Supplement: Supplementary file 1 [file Data_Sheet_1.pdf]

Supplementary material

Table of contents

Supplementary material 1

Table S1. List of nosological entities analysed in the study and corresponding codes according to the International Classification of Diseases: ICD-9, ICD-9-CM, ICD-10 2

Table S2. Results of the data quality checks of personally identifiable information for the SEBAL study cohort and the Carabinieri (CC) control cohort 3

Table S3a. Number of person-years at risk for the SEBAL and Carabinieri control cohorts in each stratum used to compute Standardised Mortality Ratio (SMR). Numbers are shown by age, area of birth and year of follow-up. The ratio between the number of person-years at risk in the Carabinieri cohort and in the SEBAL cohort (CC/SEBAL Ratio) is shown by age and area of birth 4

Table S3b. Number of person-years at risk for the SEBAL and Carabinieri control cohorts in each stratum used to compute Standardised Hospitalization Ratio (SHR). Numbers are shown by age, area of birth and year of follow-up. The ratio between the number of person-years at risk in the Carabinieri cohort and in the SEBAL cohort (CC/SEBAL Ratio) is shown by age and area of birth.. 5

Table S4. Crude and standardized (Std) mortality and hospitalization rates (x 100,000) for the SEBAL cohort and the Italian general population, with 95% confidence interval (CI). 6

Figure S1. Ratio of person-years at risk between the Carabinieri control (CC) and SEBAL cohorts by age group and year of follow-up. Subjects born in Northern/Central Italy (A) and Southern Italy/Abroad (B). Mortality study 1999-2018 ... 8

Table S5. Hospitalisation in the SEBAL cohort for all causes of disease analysed in the study, by number of missions. Number of observed and expected events in 2005-2018 using the risk profile of the **Italian population** as reference, Standardized Hospitalisation Ratio (SHR) with 95% confidence interval (95%CI). Statistically significant values in bold. 9

Table S6. Hospitalisation in the SEBAL cohort for all causes of disease analysed in the study, by number of missions. Number of observed and expected events in 2005-2018 using the risk profile of the Carabinieri control cohort as reference, Standardized Hospitalisation Ratio (SHR) with 95% confidence interval (95%CI). Statistically significant values in bold. 10

Table S1. List of nosological entities analysed in the study and corresponding codes according to the International Classification of Diseases: ICD-9, ICD-9-CM, ICD-10

Nosological entities	ICD-9/ICD-9-CM	ICD-10
**All causes**	001-629, 680-999	A00-N99, P00-T99
**All causes due to pathology**	001-629, 680-799	A00-N99, P00-R99
**Circulatory system**	390-459	I00-I99
**Digestive system**	520-579	K00-K93
**Genitourinary system**	580-629	N00-N99
**Muscoloskeletal system**	710-739	M00-M99
**Respiratory system**	460-519	J00-J99
**Infectious and parasitic diseases**	001-139	A00-B99
**Endocrine diseases**	240-259	E00-E35
**All cancers** (excluding non-melanoma skin cancer)	140-172, 174-208	C00-C43, C45-C97
**All cancers**		
**Head and Neck**	141, 143-149	C01-C06, C09-C14
**Digestive system and peritoneum**	150-159	C15-C26, C48
Oesophagus	150	C15
Stomach	151	C16
Colon-Rectum	153, 154, 159.0	C18-C21, C26.0
Liver and intrahepatic bile ducts	155	C22
Gallbladder and extrahepatic bile ducts	156	C23, C24
Pancreas	157	C25
**Respiratory and intrathoracic organs**	160-169	C30-C39
Larynx	161	C32
Lung, bronchus, trachea	162	C33, C34
**Genitourinary Organs**	185-189	C60-C68
Prostate	185	C61
Testis	186	C62
Bladder	188	C67
Kidney and other unspecified urinary organs	189	C64-C66, C68
**Lympho-haematopoietic system**	200-208	C81-C96
Leukaemias	204-208	C91-C95
Non-Hodgkin lymphoma (NHL)	200, 202	C82-C85, C96
Hodgkin lymphoma	201	C81
Multiple myeloma and immunoproliferative neoplasms	203	C88, C90
**Bones and joint cartilages**	170	C40-C41
**Connective and soft tissue**	171	C47, C49
**Skin melanoma**	172	C43
**Central Nervous System (CNS)**	191-192	C70-C72
Brain	191	C71
**Thyroid**	193	C73

Table S2. Results of the data quality checks of personally identifiable information for the SEBAL study cohort and the Carabinieri (CC) control cohort

**Exclusion criteria**	**SEBAL cohort**		**CC cohort**	
	**N**	**%**	**N**	**%**
Initial cohort (a)	71,144		114,260	
exclusions for missing tax code and full date of birth (b)	12	0.02		
exclusions for tax code incoherent with other personal data (c)	10	0.01	7	0.01
exclusions for missing and unreconstructable tax code (d)	317	0.45	414	0.36
exclusions for age <18 years or >80 years (e)	90	0.13	2	0.00
exclusions of CC subjects belonging to the SEBAL cohort (f)			6,002	5.25
**Final cohort for linkage with mortality data (a-b-e-f)**	**71,042**		**108,256**	
**Final cohort for linkage with hospitalization data (a-b-c-d-e-f)**	**70,715**		**107,835**	

Table S3a. Number of person-years at risk for the SEBAL and Carabinieri control cohorts in each stratum used to compute Standardised Mortality Ratio (SMR). Numbers are shown by age, area of birth and year of follow-up. The ratio between the number of person-years at risk in the Carabinieri cohort and in the SEBAL cohort (CC/SEBAL Ratio) is shown by age and area of birth

**Standardisation strata**		**Person-years at risk – Mortality (1999-2018)**													
		**SEBAL cohort**							**Carabinieri (CC) Control cohort**						
		**18-29**	**30-39**	**40-49**	**50-59**	**60-69**	**70-79**	**total**	**18-29**	**30-39**	**40-49**	**50-59**	**60-69**	**70-79**	**total**
**Age**		379,929	529,696	270,969	108,230	19,345	993	**1,309,162**	271,463	693,519	715,870	337,014	64,675	9,197	**2,091,738**
								**CC/SEBAL Ratio**	**0.7**	**1.3**	**2.6**	**3.1**	**3.3**	**9.3**	**1.6**
**Age-Area**	**North/Centre**	85,812	138,264	86,663	31,985	5,694	355	**348,773**	123,852	278,981	238,859	90,471	16,302	2792	**751,257**
	**South***	294,117	391,432	184,306	76,245	13,651	638	**960,389**	147,611	414,538	477,011	246,543	48,373	6,405	**1,340,481**
	**North/Centre**							**CC/SEBAL Ratio**	**1.4**	**2.0**	**2.8**	**2.8**	**2.9**	**7.9**	**2.2**
	**South***								**0.5**	**1.1**	**2.6**	**3.2**	**3.5**	**10.0**	**1.4**
**Age-Year of follow-up**	**1999**	20,380	5,932	1,731	229	4	-	**28,276**	44,064	47,804	13,507	2,843	31	6	**108,255**
	**2000**	28,921	8,799	3,305	486	11	-	**41,522**	39,499	49,089	16,230	3,200	192	4	**108,214**
	**2001**	35,624	11,172	4,762	757	22	-	**52,337**	35,130	49,491	19,487	3,625	420	3	**108,156**
	**2002**	40,293	13,377	6,358	1,066	35	-	**61,129**	30,995	49,325	23,065	4,111	587	2	**108,085**
	**2003**	41,528	15,795	7,979	1,411	53	-	**66,766**	27,085	48,363	27,088	4,712	749	2	**107,999**
	**2004**	41,122	18,307	9,618	1,803	83	-	**70,933**	23,282	46,754	31,555	5,292	1,014	3	**107,900**
	**2005**	37,837	19,887	10,802	2,248	111	-	**70,885**	19,899	43,792	36,143	6,206	1,294	2	**107,336**
	**2006**	34,360	21,744	11,811	2,780	157	-	**70,852**	16,961	40,244	39,725	7,498	1,555	3	**105,986**
	**2007**	30,492	23,975	12,760	3,349	239	-	**70,815**	14,264	37,482	42,309	9,079	1,904	11	**105,049**
	**2008**	25,332	27,533	13,557	4,011	339	2	**70,774**	10,945	36,072	44,197	10,960	2,306	21	**104,501**
	**2009**	19,122	32,116	14,330	4,684	471	4	**70,727**	6,747	35,828	45,579	13,071	2,736	29	**103,990**
	**2010**	12,791	36,889	14,821	5,506	664	11	**70,682**	2,565	35,567	46,512	15,647	3,054	176	**103,521**
	**2011**	7,498	40,469	15,415	6,365	867	21	**70,635**	26	33,843	46,556	18,661	3,450	388	**102,924**
	**2012**	3,223	42,902	15,968	7,331	1,116	34	**70,574**	1	29,913	46,129	21,958	3,926	536	**102,463**
	**2013**	1,138	42,733	16,828	8,361	1,412	50	**70,522**	0	26,198	45,083	25,626	4,492	686	**102,085**
	**2014**	247	40,674	18,223	9,499	1,755	78	**70,476**	0	22,588	43,478	29,759	5,013	933	**101,771**
	**2015**	20	37,640	19,791	10,670	2,187	103	**70,411**	0	19,472	40,818	34,085	5,860	1,188	**101,423**
	**2016**	1	34,197	21,638	11,667	2,702	145	**70,350**	0	16,731	38,025	37,779	7,102	1,413	**101,050**
	**2017**	0	30,343	23,859	12,609	3,243	223	**70,277**	0	14,120	35,713	40,491	8,609	1,723	**100,656**
	**2018**	0	25,212	27,413	13,398	3,874	322	**70,219**	0	10,843	34,671	42,411	10,381	2,068	**100,374**

*South: Southern Italy and Islands and Abroad

Table S3b. Number of person-years at risk for the SEBAL and Carabinieri control cohorts in each stratum used to compute Standardised Hospitalization Ratio (SHR). Numbers are shown by age, area of birth and year of follow-up. The ratio between the number of person-years at risk in the Carabinieri cohort and in the SEBAL cohort (CC/SEBAL Ratio) is shown by age and area of birth.

Standardisation strata		Person-years at risk – Hospitalization (2005-2018)													
		**SEBAL cohort**							**Carabinieri (CC) Control cohort**						
**Age**		**18-29**	**30-39**	**40-49**	**50-59**	**60-69**	**70-79**	**total**	**18-29**	**30-39**	**40-49**	**50-59**	**60-69**	**70-79**	**total**
		170,515	453,909	236,752	102,105	18,941	996	**983,217**	70,034	396,057	577,034	310,223	61,447	9,172	**1,423,967**
								**CC/SEBAL Ratio**	**0.4**	**0.9**	**2.4**	**3.0**	**3.2**	**9.2**	**1.4**
**Age-Area**	**North/Centre**	36,854	111,767	76,988	29,952	5,566	356	**261,483**	33,900	169,909	205,137	83,267	15,327	2,760	**510,301**
	**South***	133,661	342,142	159,764	72,153	13,375	639	**721,734**	36,134	226,148	371,897	226,956	46,120	6,412	**913,667**
	**North/Centre**							**CC/SEBAL Ratio**	**0.92**	**1.52**	**2.66**	**2.78**	**2.75**	**7.75**	**1.95**
	**South***								**0.27**	**0.66**	**2.33**	**3.15**	**3.45**	**10.03**	**1.27**
**Age-Year of follow-up**	**2005**	37,544	19,855	10,784	2,225	109	-	**70,517**	19,517	42,890	35,512	6,188	1,290	1	**105,398**
	**2006**	34,080	21,708	11,791	2,749	158	-	**70,486**	16,661	39,513	39,051	7,488	1,552	2	**104,267**
	**2007**	30,205	23,953	12,732	3,322	241	-	**70,453**	14,016	36,883	41,690	9,067	1,903	10	**103,569**
	**2008**	25,058	27,506	13,526	3,980	338	2	**70,410**	10,724	35,542	43,561	10,913	2,306	19	**103,065**
	**2009**	18,910	32,043	14,290	4,645	469	4	**70,361**	6,598	35,285	44,951	13,008	2,724	31	**102,597**
	**2010**	12,694	36,702	14,780	5,475	660	12	**70,323**	2,493	35,010	45,823	15,547	3,039	183	**102,094**
	**2011**	7,429	40,259	15,375	6,328	868	21	**70,280**	24	33,301	45,921	18,538	3,427	394	**101,606**
	**2012**	3,199	42,656	15,940	7,309	1,098	33	**70,236**	1	29,448	45,581	21,781	3,908	536	**101,255**
	**2013**	1,129	42,468	16,809	8,348	1,387	52	**70,192**	-	25,784	44,571	25,390	4,465	693	**100,903**
	**2014**	246	40,415	18,183	9,482	1,734	77	**70,138**	-	22,249	42,949	29,439	4,993	935	**100,565**
	**2015**	20	37,375	19,756	10,660	2,163	102	**70,076**	-	19,169	40,329	33,697	5,846	1,187	**100,228**
	**2016**	1	33,946	21,607	11,644	2,669	145	**70,013**	-	16,469	37,584	37,328	7,086	1,406	**99,873**
	**2017**	-	30,087	23,838	12,589	3,210	226	**69,950**	-	13,886	35,312	39,993	8,593	1,715	**99,499**
	**2018**	-	24,936	27,341	13,349	3,836	320	**69,782**	-	10,628	34,199	41,846	10,315	2,059	**99,047**

*South: Southern Italy and Islands and Abroad

Table S4. Crude and standardized (Std) mortality and hospitalization rates (x 100,000) for the SEBAL cohort and the Italian general population, with 95% confidence interval (CI).

Cause of disease	ICD-9/ ICD-9-CM	ICD-10	Mortality (1999-2018)							Hospitalization (2005-2018)						
			SEBAL cohort				Italian Population			SEBAL cohort				Italian Population		
			Crude rate	Std rate	95%CI		Crude rate	95%CI		Crude rate	Std rate	95%CI		Crude rate	95%CI	
**All causes***	001-629, 680-999	A00-N99, P00-T99	68	309	289	330	681	680	682	6,990	11,116	11,033	11,200	14,067	14,062	14,071
**All causes due to pathology***	001-629, 680-799	A00-N99, P00-R99	46	253	234	274	641	640	642	6,152	10,382	10,300	10,465	12,898	12,894	12,902
**Circulatory system**	390-459	I00-I99	11	71	60	83	193	193	194	610	1,264	1,232	1,297	1,414	1,413	1,416
**Digestive system**	520-579	K00-K93	2	8	5	12	33	32	33	833	1,157	1,132	1,184	1,322	1,320	1,323
**Genitourinary system**	580-629	N00-N99	0	1	0	4	8	8	8	341	602	582	623	686	685	687
**Muscoloskeletal system**	710-739	M00-M99	0	0	0	2	2	2	2	862	1,136	1,111	1,161	895	894	896
**Respiratory system**	460-519	J00-J99	1	7	4	13	37	36	37	397	591	572	610	660	659	661
**Infectious and parasitic diseases**	001-139	A00-B99	1	2	1	4	12	12	12	86	99	92	106	182	182	183
**Endocrine diseases**	240-259	E00-E35	1	4	2	9	21	21	21	62	102	95	111	143	142	143
**All cancers** (excluding non-melanoma skin cancer)	140-172, 174-208	C00-C43, C45-C97	26	156	140	173	280	280	281	162	592	563	622	722	721	723
**All cancers**																
Head and Neck	***141, 143-149***	***C01-C06, C09-C14***	0.8	3.1	1.5	5.6	11.4	11.3	11.5	3.2	8.8	6.0	12.4	16.5	16.4	16.7
**Digestive system and peritoneum**	***150-159***	***C15-C26, C48***	8.2	52.4	42.9	63.3	101.7	101.4	102.0	28.7	133.3	118.2	149.7	180.4	179.9	180.9
Oesophagus	*150*	*C15*	0.4	2.3	0.7	5.3	4.9	4.9	5.0	0.9	3.9	1.8	7.4	6.6	6.5	6.7
Stomach	*151*	*C16*	1.9	11.9	7.7	17.6	18.0	17.9	18.2	4.6	22.0	16.0	29.4	26.5	26.3	26.7
Colon-Rectum	*153-154, 159.0*	*C18-C21, C26.0*	3.1	20.5	14.6	27.9	35.3	35.2	35.5	16.1	77.3	65.7	90.2	89.3	89.0	89.6
Liver and intrahepatic bile ducts	*155*	*C22*	0.6	4.0	1.7	7.9	21.5	21.3	21.6	2.2	10.3	6.5	15.7	32.0	31.8	32.2
Gallbladder and extrahepatic bile ducts	*156*	*C23, C24*	0.4	2.9	0.9	6.7	4.0	4.0	4.1	0.8	4.2	1.8	8.2	7.7	7.6	7.8
Pancreas	*157*	*C25*	1.5	8.5	5.1	13.2	16.4	16.3	16.6	2.8	12.4	8.3	18.0	19.9	19.8	20.1
**Respiratory and intrathoracic organs**	***160-169***	***C30-C39***	4.7	35.2	26.9	45.2	91.3	91.0	91.6	12.7	66.7	55.5	79.5	115.7	115.3	116.1
Larynx	*161*	*C32*	0.0	0.0		1.7	5.1	5.0	5.1	1.8	7.0	4.1	11.0	16.8	16.6	16.9
Lung, bronchus, trachea	*162*	*C33, C34*	4.4	33.8	25.6	43.9	84.4	84.1	84.6	8.7	52.7	42.1	65.1	92.0	91.6	92.3
**Genitourinary organs**	***185-189***	***C60-C68***	1.9	17.6	11.4	25.9	33.7	33.5	33.8	55.1	234.5	215.1	255.1	247.8	247.2	248.4
Prostate	*185*	*C61*	0.3	5.8	1.6	14.8	14.3	14.1	14.4	14.7	121.9	102.9	143.5	117.2	116.9	117.6
Testis	*186*	*C62*	0.3	0.2	0.1	0.5	0.3	0.3	0.3	16.0	10.3	8.7	12.0	10.5	10.4	10.6
Bladder	*188*	*C67*	0.4	4.1	1.3	9.6	10.8	10.7	10.9	16.1	84.0	71.4	98.1	93.3	92.9	93.6

Cause of disease	ICD-9/ ICD-9-CM	ICD-10	Mortality (1999-2018)							Hospitalization (2005-2018)						
			SEBAL cohort				Italian Population			SEBAL cohort				Italian Population		
			Crude rate	Std rate	95%CI		Crude rate	95%CI		Crude rate	Std rate	95%CI		Crude rate	95%CI	
Kidney and other unspecified urinary organs	*189*	*C64-C66, C68*	0.9	5.5	2.8	9.6	7.9	7.8	8.0	8.5	28.9	23.0	35.8	35.5	35.2	35.7
**Lympho-haematopoietic system**	***200-208***	***C81-C96***	3.5	14.8	10.9	19.8	22.4	22.2	22.5	23.5	51.0	44.6	58.0	58.7	58.4	59.0
Leukaemias	*204-208*	*C91-C95*	1.7	6.6	4.1	10.0	9.1	9.0	9.2	7.0	17.0	13.3	21.6	18.6	18.4	18.7
Non-Hodgkin lymphoma (NHL)	*200, 202*	*C82-C85,C96*	1.1	4.7	2.6	7.8	7.8	7.7	7.9	12.7	26.2	21.8	31.2	29.3	29.1	29.5
Hodgkin lymphoma	*201*	*C81*	0.4	0.7	0.2	1.5	0.8	0.7	0.8	3.3	3.0	2.1	4.3	4.9	4.8	5.0
Multiple myeloma and immunoproliferative neoplasms	*203*	*C88, C90*	0.3	2.3	0.6	6.0	4.7	4.6	4.7	2.0	8.1	5.0	12.5	9.8	9.7	9.9
Bone and joint cartilages	*170*	*C40-C41*	0.2	0.3	0.0	1.0	0.8	0.8	0.8	1.1	1.8	0.9	3.2	3.1	3.0	3.1
Connective and soft tissue	*171*	*C47, C49*	0.6	1.4	0.6	2.7	1.4	1.4	1.4	3.4	6.4	4.4	8.9	6.3	6.2	6.4
Skin melanoma	172	C43	1.5	3.9	2.3	6.1	3.4	3.4	3.5	9.5	18.6	15.1	22.8	14.1	14.0	14.3
**Central Nervous System (CNS)**	***191-192***	***C70-C72***	2.7	6.9	4.8	9.6	7.6	7.5	7.7	6.7	13.7	10.6	17.5	16.3	16.2	16.5
Brain	*191*	*C71*	2.6	6.7	4.6	9.4	7.4	7.3	7.5	6.1	12.5	9.5	16.1	15.1	15.0	15.3
Thyroid	*193*	*C73*	0.2	0.7	0.1	2.6	0.7	0.7	0.7	14.4	16.5	13.9	19.4	11.9	11.7	12.0

* including multiple hospidalizations

Figure S1. Ratio of person-years at risk between the Carabinieri control (CC) and SEBAL cohorts by age group and year of follow-up. Subjects born in Northern/Central Italy (A) and Southern Italy/Abroad (B). Mortality study 1999-2018

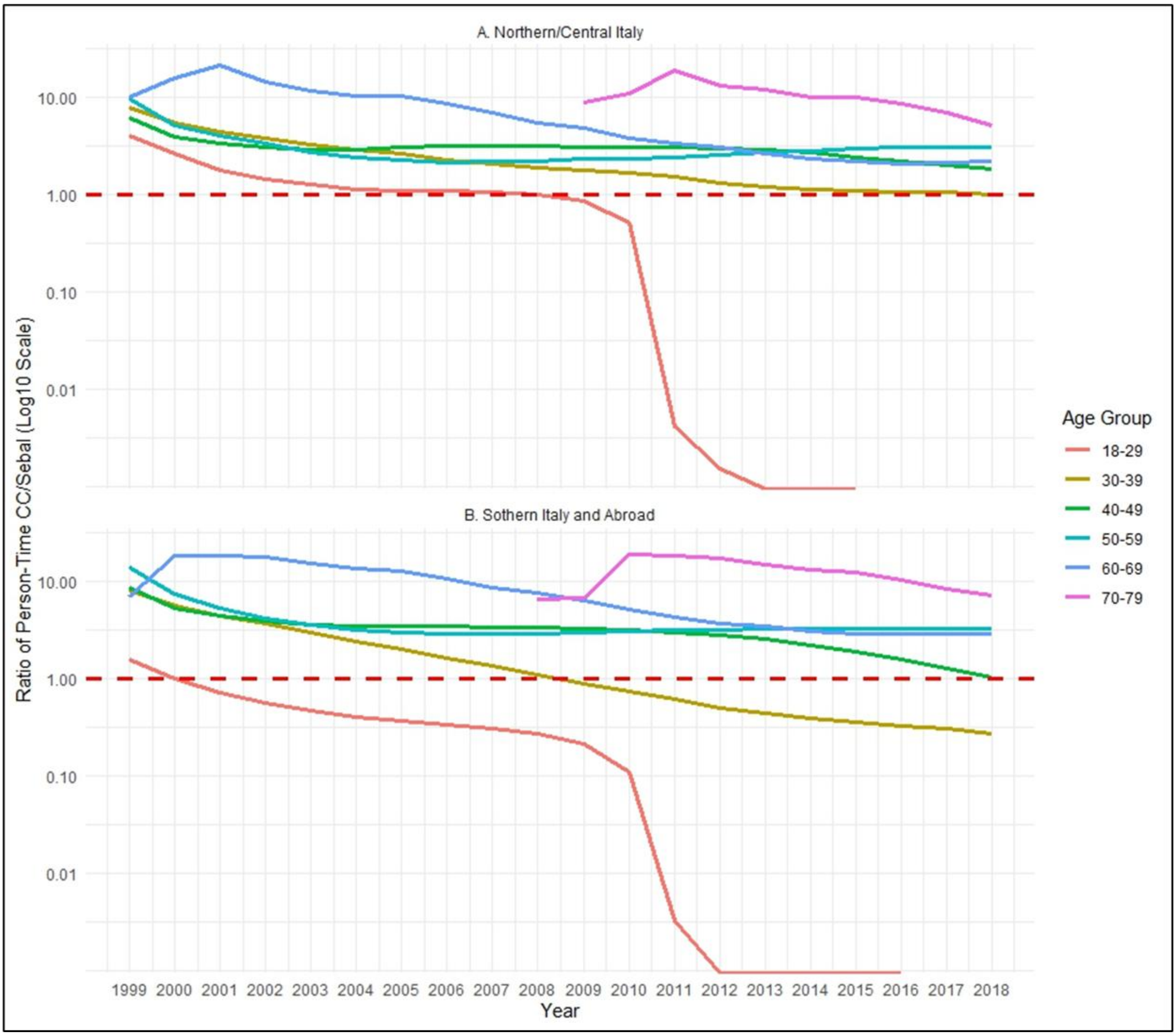

Table S5. Hospitalisation in the SEBAL cohort for all causes of disease analysed in the study, by number of missions. Number of observed and expected events in 2005-2018 using the risk profile of the **Italian population** as reference, Standardized Hospitalisation Ratio (SHR) with 95% confidence interval (95%CI). Statistically significant excess relative risks are in bold.

Cause of disease	ONLY ONE MISSION					MULTIPLE MISSIONS				
	Observed Events	Expected Events	SHR	95%CI		Observed Events	Expected Events	SHR	95%CI	
**All causes**	27,241	32,175	0.85	0.84	0.86	41,471	54,795	0.76	0.75	0.76
**All causes due to pathology**	24,008	27,842	0.86	0.85	0.87	36,483	47,324	0.77	0.76	0.78
**Circulatory system**	2,229	2,387	0.93	0.90	0.97	3,522	4,048	0.87	0.84	0.90
**Digestive system**	2,903	3,227	0.90	0.87	0.93	4,825	5,598	0.86	0.84	0.89
**Genitourinary system**	1,269	1,392	0.91	0.86	0.96	2,014	2,347	0.86	0.82	0.90
**Muscoloskeletal system**	2,827	2,340	**1.21**	**1.16**	**1.25**	5,159	3,955	**1.30**	**1.27**	**1.34**
**Respiratory system**	1,465	1,582	0.93	0.88	0.97	2,332	2,656	0.88	0.84	0.91
**Infectious and parasitic diseases**	336	567	0.59	0.53	0.66	506	983	0.51	0.47	0.56
**Endocrine diseases**	238	316	0.75	0.66	0.85	375	538	0.70	0.63	0.77
**All malignant cancers** (excluding non-melanoma skin cancer)	651	742	0.88	0.81	0.95	928	1,182	0.78	0.74	0.84
**Head and Neck**	12	23	0.52	0.27	0.91	20	37	0.54	0.33	0.83
**Digestive system and peritoneum**	132	152	0.87	0.73	1.03	151	231	0.65	0.55	0.77
Oesophagus	5	6	0.84	0.27	1.96	4	9	0.43	0.12	1.11
Stomach	19	21	0.89	0.54	1.39	26	33	0.79	0.52	1.16
Colon-Rectum	80	73	1.09	0.87	1.36	79	111	0.71	0.57	0.89
Liver and intrahepatic bile ducts	9	27	0.33	0.15	0.63	13	41	0.32	0.17	0.54
Gallbladder and extrahepatic bile ducts	2	6	0.34	0.04	1.22	6	9	0.69	0.25	1.49
Pancreas	14	18	0.79	0.43	1.33	14	27	0.52	0.28	0.87
**Respiratory and intrathoracic organs**	53	88	0.60	0.45	0.79	72	129	0.56	0.44	0.70
Larynx	8	17	0.46	0.20	0.92	10	26	0.38	0.18	0.70
Lung, bronchus, trachea	37	62	0.60	0.42	0.83	49	88	0.55	0.41	0.73
**Genitourinary Organs**	222	225	0.98	0.86	1.12	320	347	0.92	0.82	1.03
Prostate	67	60	1.11	0.86	1.41	78	79	0.98	0.78	1.23
Testis	56	59	0.95	0.72	1.24	102	103	0.99	0.81	1.20
Bladder	69	71	0.97	0.76	1.23	90	106	0.85	0.68	1.05
Kidney and other unspecified urinary organs	29	39	0.74	0.49	1.06	55	64	0.86	0.65	1.12
**Lympho-haematopoietic system**	95	100	0.95	0.77	1.16	136	166	0.82	0.69	0.97
Leukaemias	23	28	0.81	0.51	1.22	46	47	0.98	0.72	1.31
Non-Hodgkin lymphoma (NHL)	55	52	1.05	0.79	1.37	70	88	0.80	0.62	1.01
Hodgkin lymphoma	14	20	0.71	0.39	1.20	19	34	0.56	0.34	0.88
Multiple myeloma and immunoproliferative neoplasms	10	9	1.06	0.51	1.95	10	15	0.68	0.33	1.25
**Bones and joint cartilages**	4	7	0.57	0.16	1.46	7	12	0.60	0.24	1.23
**Connective and soft tissue**	12	12	0.96	0.50	1.68	22	21	1.05	0.66	1.59
**Skin melanoma**	48	26	**1.82**	**1.34**	**2.42**	46	45	1.02	0.75	1.37
**Central Nervous System (CNS)**	24	29	0.81	0.52	1.21	42	49	0.86	0.62	1.16
Brain	20	27	0.73	0.45	1.13	40	45	0.88	0.63	1.20
**Thyroid**	45	37	1.21	0.89	1.63	97	65	**1.49**	**1.21**	**1.82**

Table S6. Hospitalisation in the SEBAL cohort for all causes of disease analysed in the study, by number of missions. Number of observed and expected events in 2005-2018 using the risk profile of the Carabinieri control cohort as reference, Standardized Hospitalisation Ratio (SHR) with 95% confidence interval (95%CI). Statistically significant excess relative risks are in bold.

Cause of disease	ONLY ONE MISSION					MULTIPLE MISSIONS				
	Observed Events	Expected Events	SHR	95%CI		Observed Events	Expected Events	SHR	95%CI	
**All causes**	27,241	31,630	0.86	0.85	0.87	41,471	54,076	0.77	0.76	0.77
**All causes due to pathology**	24,008	27,925	0.86	0.85	0.87	36,483	47,767	0.76	0.76	0.77
**Circulatory system**	2,229	2,833	0.79	0.75	0.82	3,522	4,913	0.72	0.69	0.74
**Digestive system**	2,903	3,574	0.81	0.78	0.84	4,825	6,256	0.77	0.75	0.79
**Genitourinary system**	1,269	1,467	0.86	0.82	0.91	2,014	2,509	0.80	0.77	0.84
**Muscoloskeletal system**	2,827	2,985	0.95	0.91	0.98	5,159	5,071	1.02	0.99	1.05
**Respiratory system**	1,465	1,630	0.90	0.85	0.95	2,332	2,775	0.84	0.81	0.88
**Infectious and parasitic diseases**	336	379	0.89	0.79	0.99	506	644	0.79	0.72	0.86
**Endocrine diseases**	238	365	0.65	0.57	0.74	375	647	0.58	0.52	0.64
**All malignant cancers** (excluding non-melanoma skin cancer)	651	670	0.97	0.90	1.05	928	1,070	0.87	0.81	0.92
**Head and Neck**	12	18	0.66	0.34	1.14	20	31	0.64	0.39	0.99
**Digestive system and peritoneum**	132	144	0.92	0.77	1.09	151	221	0.68	0.58	0.80
Oesophagus	5	5	0.98	0.32	2.29	4	8	0.53	0.14	1.35
Stomach	19	17	1.14	0.69	1.79	26	26	0.98	0.64	1.44
Colon-Rectum	80	81	0.99	0.79	1.23	79	123	0.64	0.51	0.80
Liver and intrahepatic bile ducts	9	15	0.59	0.27	1.12	13	23	0.58	0.31	0.99
Gallbladder and extrahepatic bile ducts	2	6	0.33	0.04	1.20	6	9	0.65	0.24	1.42
Pancreas	14	18	0.79	0.43	1.33	14	27	0.52	0.28	0.87
**Respiratory and intrathoracic organs**	53	62	0.85	0.64	1.11	72	93	0.77	0.60	0.97
Larynx	8	12	0.69	0.30	1.36	10	18	0.55	0.27	1.02
Lung, bronchus, trachea	37	42	0.87	0.61	1.20	49	62	0.79	0.58	1.04
**Genitourinary Organs**	222	233	0.95	0.83	1.08	320	359	0.89	0.80	0.99
Prostate	67	67	1.00	0.77	1.27	78	90	0.87	0.69	1.09
Bladder	69	65	1.05	0.82	1.33	90	99	0.91	0.73	1.12
Kidney and other unspecified urinary organs	29	43	0.67	0.45	0.97	55	70	0.78	0.59	1.02
**Lympho-haematopoietic system**	95	81	1.17	0.95	1.43	136	132	1.03	0.86	1.22
Leukaemias	23	25	0.91	0.58	1.37	46	42	1.10	0.81	1.47
Non-Hodgkin lymphoma (NHL)	55	42	1.32	0.99	1.72	70	68	1.03	0.80	1.30
Multiple myeloma and immunoproliferative neoplasms	10	7	1.38	0.66	2.53	10	12	0.82	0.39	1.51
